# Supplementary material for: Phenology is the dominant control of methane emissions in a tropical non-forested wetland
Source: Nat Commun. 2022 Jan 10;13:133. doi: 10.1038/s41467-021-27786-4 (PMC8748800; doi:10.1038/s41467-021-27786-4)
Supplement: Supplementary file 1 — Supplementary Information [file 41467_2021_27786_MOESM1_ESM.pdf]

Phenology is the dominant control of methane emissions in a tropical non-forested wetland

*Carole Helfter[1], Mangaliso Gondwe[2], Michael Murray-Hudson[2], Anastacia Makati[2], Mark F. Lunt[3], Paul I. Palmer[3,4] and Ute Skiba[1].

[1]UK Centre for Ecology and Hydrology, Penicuik, EH26 0QB, UK.

[2]Okavango Research Institute, University of Botswana, Maun, Botswana.

[3]School of GeoSciences, University of Edinburgh, Edinburgh, UK.

[4]National Centre for Earth Observation, University of Edinburgh, Edinburgh, UK.

* Corresponding author; email: caro2@ceh.ac.uk

Supplementary information

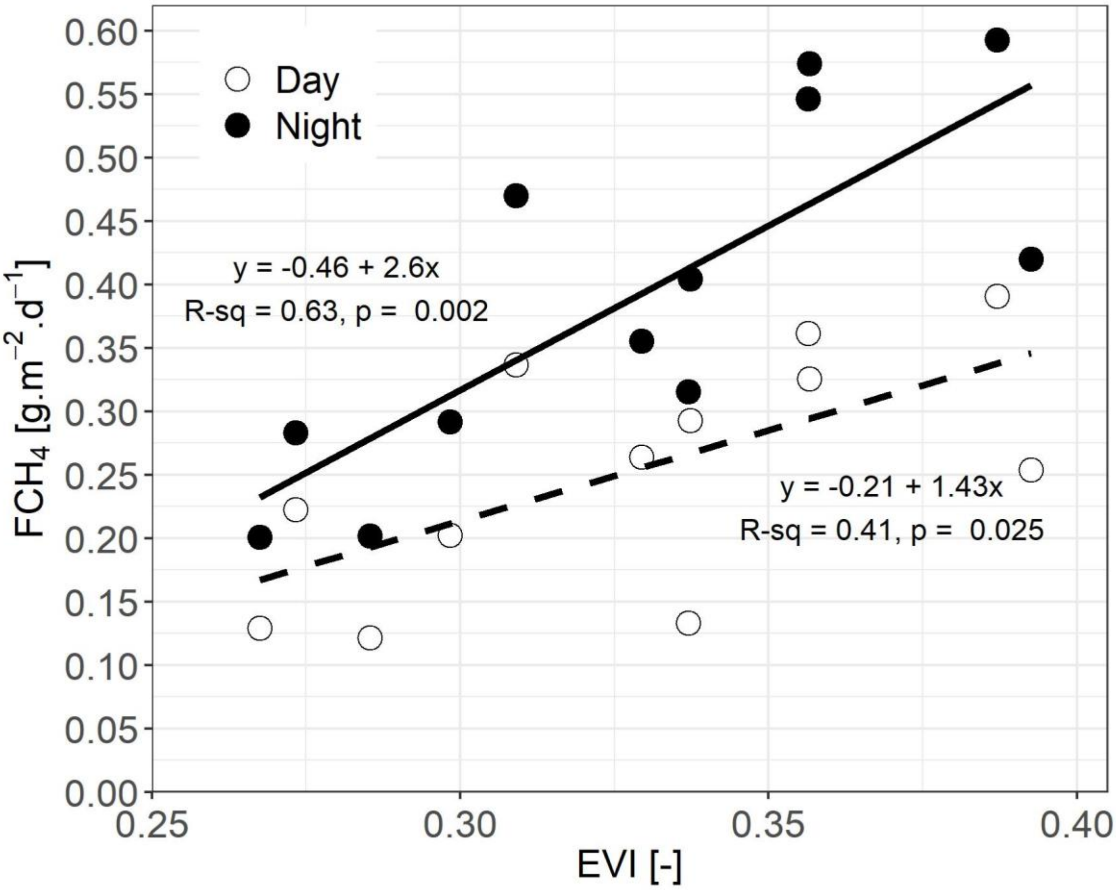

Supplementary Figure 1: Monthly median (all data years used) fluxes of CH_4 measured over a papyrus stand at Guma Lagoon (18°57'53.01"S; 22°22'16.20"E) during daytime (07:00 – 18:00, open symbols) and night time (19:00 – 06:00, solid symbols), as a function of monthly median enhanced vegetation index (EVI). The solid and dashed lines represent linear regressions for night and day data (equations and t-test two-sided p-value given in the panels): standard error of ± 0.54 and 0.18 (day), and ± 0.64 and 0.21 (night) for slope and intercept, respectively.

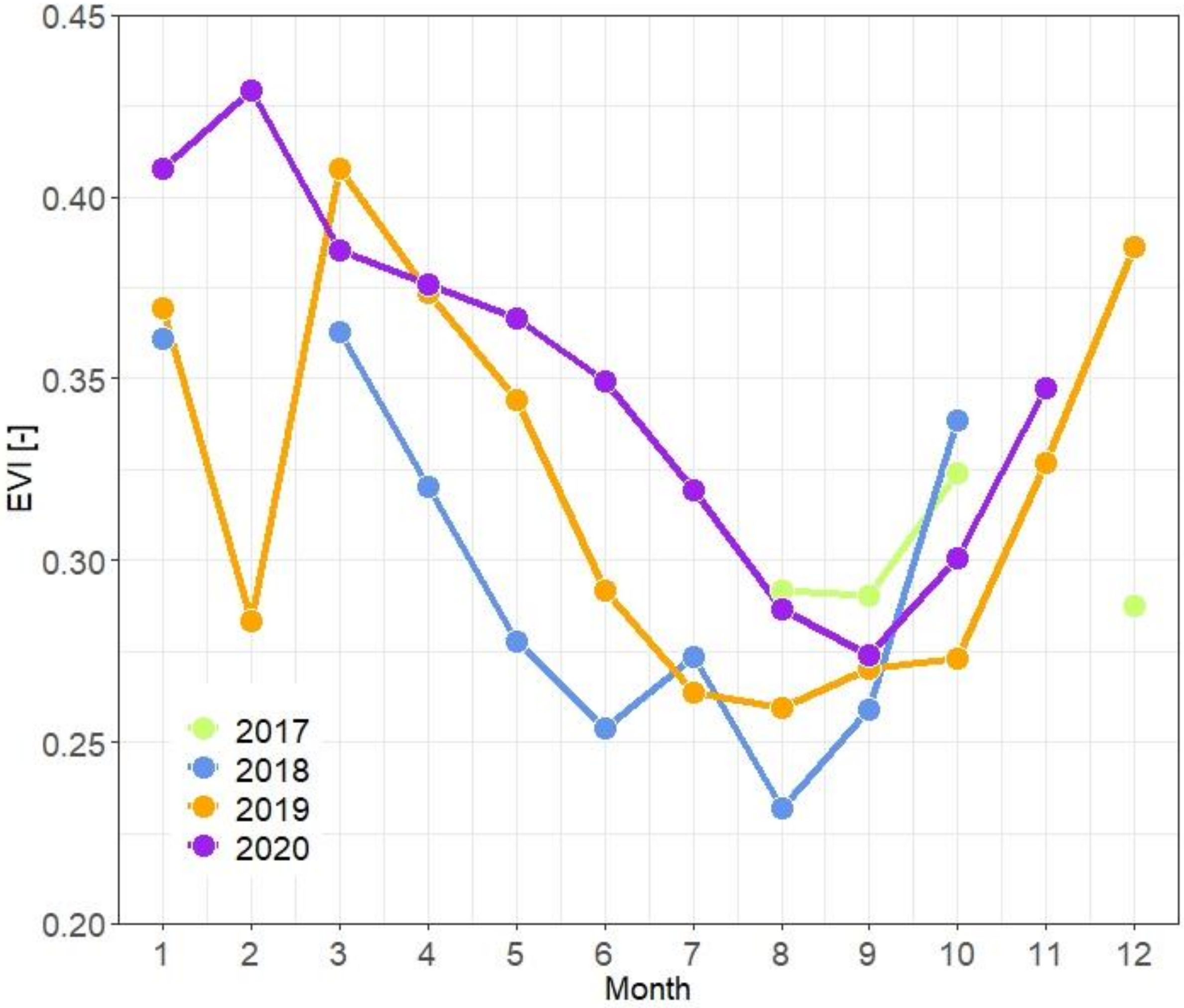

Supplementary Figure 2: Monthly mean values of the enhanced vegetation index (EVI) of the papyrus stand studied at Guma Lagoon (18°57'53.01"S; 22°22'16.20"E) for the period August 2017 – December 2020.

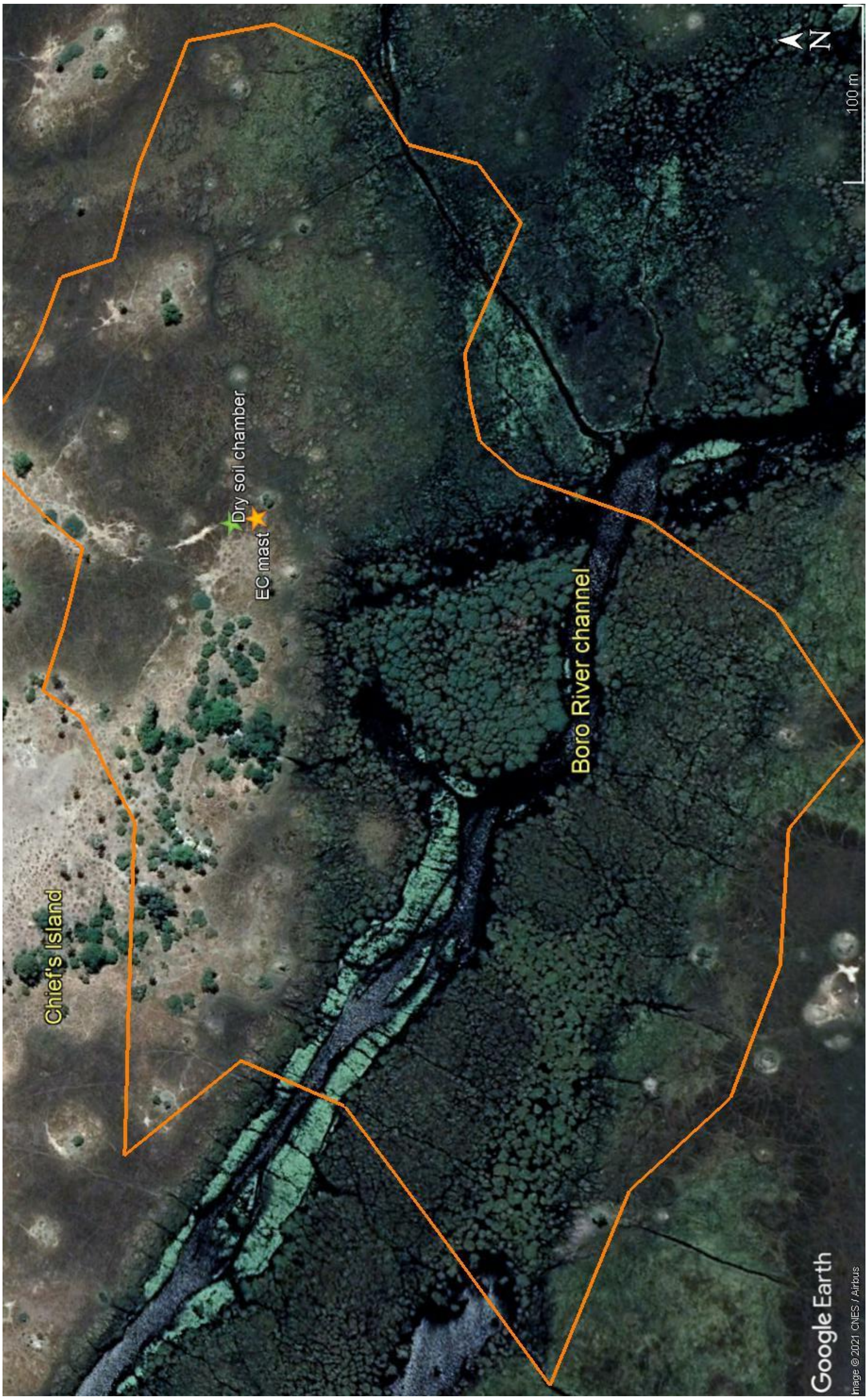

Supplementary Figure 3: satellite image of the seasonal floodplain measurement site at Nxaraga (19°32'53'' S; 23°10'45'' E). The location of the eddy-covariance (EC) mast is indicated by an orange star, and a green star marks the location of the dry soil chamber used to measure methane oxidation fluxes. The orange polygon represents the median flux footprint of the EC mast.
