# Supplementary material for: Phenology is the dominant control of methane emissions in a tropical non-forested wetland
Source: Nat Commun. 2022 Jan 10;13:133. doi: 10.1038/s41467-021-27786-4 (PMC8748800; doi:10.1038/s41467-021-27786-4)
Supplement: Supplementary file 3 — Reporting Summary [file 41467_2021_27786_MOESM3_ESM.pdf]

nature portfolio

Corresponding author(s): Carole Helfter

Last updated by author(s): Nov 18, 2021

Reporting Summary

Nature Portfolio wishes to improve the reproducibility of the work that we publish. This form provides structure for consistency and transparency in reporting. For further information on Nature Portfolio policies, see our Editorial Policies and the Editorial Policy Checklist.

Statistics

For all statistical analyses, confirm that the following items are present in the figure legend, table legend, main text, or Methods section.

n/a	Confirmed	
☐	☒	The exact sample size (n) for each experimental group/condition, given as a discrete number and unit of measurement
☒	☐	A statement on whether measurements were taken from distinct samples or whether the same sample was measured repeatedly
☐	☒	The statistical test(s) used AND whether they are one- or two-sided<br>*Only common tests should be described solely by name; describe more complex techniques in the Methods section.*
☒	☐	A description of all covariates tested
☐	☒	A description of any assumptions or corrections, such as tests of normality and adjustment for multiple comparisons
☐	☒	A full description of the statistical parameters including central tendency (e.g. means) or other basic estimates (e.g. regression coefficient) AND variation (e.g. standard deviation) or associated estimates of uncertainty (e.g. confidence intervals)
☐	☒	For null hypothesis testing, the test statistic (e.g. F, t, r) with confidence intervals, effect sizes, degrees of freedom and P value noted<br>*Give P values as exact values whenever suitable.*
☒	☐	For Bayesian analysis, information on the choice of priors and Markov chain Monte Carlo settings
☒	☐	For hierarchical and complex designs, identification of the appropriate level for tests and full reporting of outcomes
☒	☐	Estimates of effect sizes (e.g. Cohen's d, Pearson's r), indicating how they were calculated

Our web collection on statistics for biologists contains articles on many of the points above.

Software and code

Policy information about availability of computer code

Data collection	The eddy-covariance and meteorological data were logged by Campbell Scientific CR3000 dataloggers running site-and instrumentation-specific, custom programs written in CR basic (Campbell Scientific proprietary language, version 3.6).
Data analysis	The raw eddy-covariance data were processed into half-hourly fluxes using LI-COR EddyProTM (version 7.0.6) free software. The flux data were further analysed and plotted in R (version 4.0.3). The R code made use of standard statistics (e.g. mean, standard deviation) described in the manuscript. Sentinel-2 imagery were processed using SNAP (ESA Sentinel Application Platform v8.0, http://step.esa.int).

For manuscripts utilizing custom algorithms or software that are central to the research but not yet described in published literature, software must be made available to editors and reviewers. We strongly encourage code deposition in a community repository (e.g. GitHub). See the Nature Portfolio guidelines for submitting code & software for further information.

Data

Policy information about availability of data

All manuscripts must include a data availability statement. This statement should provide the following information, where applicable:

- Accession codes, unique identifiers, or web links for publicly available datasets
- A description of any restrictions on data availability
- For clinical datasets or third party data, please ensure that the statement adheres to our policy

The eddy-covariance and meteorological data generated in this study for the Guma Lagoon perennial wetland have been deposited with the UK Environmental Information Data Centre [https://doi.org/10.5285/d366ed40-af8c-42be-86f2-bb90b11a659e].
The eddy-covariance and meteorological data generated in this study for the for Nxaraga seasonal floodplain have been deposited with the UK Environmental Information Data Centre [https://doi.org/10.5285/2170ebd0-7e6f-4871-97d9-1d42e210468f].

Both datasets are publicly available under the terms of the Open Government License (https://www.nationalarchives.gov.uk/doc/open-government-licence/version/3/).
Sentinel-2 imagery was obtained from Copernicus Open Access Hub (http://scihub.copernicus.eu).

Field-specific reporting

Please select the one below that is the best fit for your research. If you are not sure, read the appropriate sections before making your selection.

☐ Life sciences ☐ Behavioural & social sciences ☒ Ecological, evolutionary & environmental sciences

For a reference copy of the document with all sections, see nature.com/documents/nr-reporting-summary-flat.pdf

Ecological, evolutionary & environmental sciences study design

All studies must disclose on these points even when the disclosure is negative.

Study description

3 years of eddy-covariance measurements of methane and carbon dioxide fluxes at two contrasting wetlands (seasonally- and perenially-flooded) in the Okavango Delta, Botswana.

Research sample

We targeted 2 contrasting wetlands to study differences in environmental controls and temporal dynamics of methane fluxes. The resulting datasets consist of continuous time series at half-hourly intervals of fluxes of methane (CH4) and carbon dioxide (CO2) resulting from land-atmosphere exchange of these gases. At the perennial swamp we studied fluxes from a floating Cyperus papyrus mat, because this species is conspicuous in permanently inundated areas of the Okavango Delta and other parts of sub-Saharan Africa. Seasonal floodplains can make up to 2/3 of the total inundated area at peak flood, but due to marked differences in hydrology and vegetation cover (typically mixed grasses), we also expected differences in land-atmosphere exchange of CH4 and CO2. The study sites we chose were hence representative of the 2 main ecohydrological zones of the Okavango Delta.

Sampling strategy

The eddy-covariance technique used in this study yields spatially-integrated flux data across variable footprints (i.e. spatial source areas) upwind of the measurement mast. The location of the mast and the physical dimensions of the setup were chosen so as to sample flux data from areas of interest in terms of vegetation cover and inundation. Specifically, we targeted a floating papyrus mat at the perennial wetland, and a uniform area of mixed grasses at the seasonal floodplain.

Data collection

The eddy-covariance instrumentation consisted of a Campbell Scientific IRGASON and a LI-COR 7700 open-path methane (CH4) analyser. The IRGASON consists of a 3D ultrasonic anemometer and open-path infrared gas analyser, providing co-located measurements of the wind vector and mass densities of carbon dioxide (CO2) and water vapour. The IRGASON was oriented into the prevailing wind direction at each site, and the LI-COR 7700 CH4 analyser was mounted onto a horizontal boom, 0.3 m from the anemometer in the crosswind plane. A Vaisala WXT520 weather station recorded air temperature, pressure, relative humidity, wind speed and direction. Total solar radiation and photosynthetically active radiation (PAR) were measured by a Skye Instruments pyranometer (model SKS1110) and quantum detector (model SKP215), respectively. A Campbell Scientific CR3000 datalogger logged all sensors (sampling rate of 10 Hz for the EC variables and 10-s interval for the meteorological parameters) using a custom data acquisition program written in CR Basic (version 3.7).
At Guma lagoon, the instrumentation was mounted onto a 3-m high tripod, which was itself located on a 3-m high platform (effective measurement height 5.5 m). The EC system was installed on land, ca. 30 meters to the west of a predominantly floating papyrus (Cyperus papyrus) mat. The papyrus mat was partially grounded to the West, where it met the shore of Guma Lagoon and it extended ca. 300 m into the lagoon in an easterly direction. The canopy height was on average 2.5 meters above water level.
At Nxaraga, a 2.5-m high EC mast was erected on the SW edge of Chief's Island to sample greenhouse gas (GHG) fluxes from the seasonal floodplain, which extends several hundred meters to the W, S and SW. The portion of floodplain within the flux footprint of the EC mast was bounded by a permanent, meandering water channel fringed by reeds and grasses such as Phragmites spp and Miscanthus junceus. The vegetation of the floodplain, which is dominated by grasses (e.g., Panicum repens, Cynodon dactylon, Sporobolus spicatus), attracts many types of herbivores (e.g. impala, buffalo) and is grazed for most of the year.
The instrumentation operated autonomously; Dr Mangaliso Gondwe from the Okavango Research Institute, University of Botswana carried out monthly site visits to service the instrumentation and collect the data.

Timing and spatial scale

Sampling was continuous over the period August 2017 - April 2021. The raw data was logged at 10 Hz and fluxes were calculated for half-hourly time intervals, which is a customary time step for eddy-covariance studies. Spatial scale: the typical flux footprint at both eddy-covariance sites extended 200-300 meters upwind from the instrument masts.

Data exclusions

Half-hourly flux data were rejected from analysis if any of the following criteria was fulfilled:
• Failure of the micrometeorological data quality controls based on the assessment of steady state conditions and integral turbulence characteristics (flag value of 2, following the 0-1-2 flagging system proposed by Foken et al.**).
• Friction velocity (u*) < 0.2 m.s-1.
• Signal strength of the LI-COR 7700 open-path CH4 analyser < 10%.
• Carbon dioxide fluxes outside the range [-40, 40] µmol.m-2.s-1.
• Methane fluxes outside the range [-50, 1000] nmol.m-2.s-1.
• Latent or sensible heat fluxes outside the range [-250, 800] W.m-2.
• Wind blowing from outside the sector [60°, 170°] at Guma Lagoon and [100°, 170°] at Nxaraga.

** Foken, T., et al., Post-field quality control, in Handbook of micrometeorology: A guide for surface flux measurements, X. Lee, Editor, Kluwer Academic: Dordrecht. p. 81-108 (2004).

Foken, T. and B. Wichura, Tools for quality assessment of surface-based flux measurements. Agr Forest Meteorol 78(1-2): 83-105 (1996).

Reproducibility	This study was conducted in real time in a natural environment subject to variable environmental conditions to assess the natural temporal variability and controls of methane fluxes. The study did not have controlled conditions (such as in e.g. clinical trials) and the concept of reproducibility is hence not applicable.
Randomization	The eddy-covariance measurement techniques yields spatially-integrated fluxes: at Guma Lagoon (perennial swamp) the vegetation of interest was Cyperus papyrus - chosen because it is a conspicuous species in African wetlands - the portion of the papyrus stand within the flux footprint of the mast consisted of juvenile, mature and senescing plants (in varying proportion over the course of the phenological cycle) and was deemed representative of more extensive papyrus beds. At Nxaraga seasonal floodplain, the flux footprint entrained areas of mixed vegetation, predominantly grasses, typical of seasonal floodplains in the Okavango Delta. The randomisation in the ecosystems studied comes therefore from the natural variability found on the ground.
Blinding	This study is not a clinical trial, nor does it involved human subjects, and the concept of blinding is therefore not relevant.

Did the study involve field work? ☒ Yes ☐ No

Field work, collection and transport

Field conditions	During the study period (August 2017 – April 2021) the mean annual temperature was 26.8 ± 3.4 °C (minimum 14.5 ± 4.1 °C and maximum 35.1 ± 3.7 °C) at Guma and 25.3 ± 2.6 °C (minimum 13.7 ± 5.3 °C and maximum 34.8 ± 3.2 °C) at Nxaraga. July was the coldest month of the year (20.7 ± 4.8 °C and 21.0 ± 6.0 °C at Guma and Nxaraga, respectively) and October the hottest (28.7 ± 6.9 °C and 28.1± 6.3 °C at Guma and Nxaraga, respectively).<br><br>Annual rainfall, measured at Guma Lagoon, was variable and ranged from 194 mm in 2019 to 714 mm in 2020.
Location	Guma Lagoon (perennial swamp): 18°57'53.01"S; 22°22'16.20" E, elevation 983 m above sea level, maximum water depth 5-6 meters.<br><br>Nxaraga (seasonal floodplain): 19°32'53'' S; 23°10'45'' E, elevation 952 m above sea level, water depth in the floodplain ranging from 0 - 1 m due to the seasonal nature of the inundation.
Access & import/export	The research was carried out under research permit EWT 8/36/4 issued on February 8 2017 by the Ministry of Environment, Natural Resources Conservation and Tourism of the Republic of Botswana.<br>No samples were exported.
Disturbance	The study caused no disturbance to the natural ecosystems. Travel to the Guma Lagoon site was mainly by road and over sand following existing tracks. Travel to Nxaraga seasonal floodplain was by boat or over land, depending on the level of inundation. Travel and field assistance was provided by experienced research assistants from the Okavango Research Institute (ORI), University of Botswana, who possess extensive knowledge of the fauna, flora and all aspects of the local environment. No permanent structures were erected and the instrumentation was installed on temporary tripods under the guidance of ORI staff.

Reporting for specific materials, systems and methods

We require information from authors about some types of materials, experimental systems and methods used in many studies. Here, indicate whether each material, system or method listed is relevant to your study. If you are not sure if a list item applies to your research, read the appropriate section before selecting a response.

Materials & experimental systems

n/a	Involved in the study	
☒	☐	Antibodies
☒	☐	Eukaryotic cell lines
☒	☐	Palaeontology and archaeology
☒	☐	Animals and other organisms
☒	☐	Human research participants
☒	☐	Clinical data
☒	☐	Dual use research of concern

Methods

n/a	Involved in the study	
☒	☐	ChIP-seq
☒	☐	Flow cytometry
☒	☐	MRI-based neuroimaging
